# Hidden Attractors in Discrete Dynamical Systems

**DOI:** 10.3390/e23050616

**Published:** 2021-05-16

**Authors:** Marek Berezowski, Marcin Lawnik

**Affiliations:** 1 Faculty of Chemical Engineering and Technology, Cracow University of Technology, ul. Warszawska 24, 30-155 Kraków, Poland; 2 Department of Mathematics Applications and Methods for Artificial Intelligence, Silesian University of Technology, Kaszubska 23, 44-100 Gliwice, Poland; marcin.lawnik@polsl.pl

**Keywords:** chaos, discrete system, dynamics, fractal, hidden attractor

## Abstract

Research using chaos theory allows for a better understanding of many phenomena modeled by means of dynamical systems. The appearance of chaos in a given process can lead to very negative effects, e.g., in the construction of bridges or in systems based on chemical reactors. This problem is important, especially when in a given dynamic process there are so-called hidden attractors. In the scientific literature, we can find many works that deal with this issue from both the theoretical and practical points of view. The vast majority of these works concern multidimensional continuous systems. Our work shows these attractors in discrete systems. They can occur in Newton’s recursion and in numerical integration.

## 1. Introduction

In nature, many phenomena are generated by dynamical systems. These are, i.a., fixed points, periodic or non-periodic (quasiperiodic) oscillations, as well as chaotic oscillations. From a scientific and practical point of view, chaotic oscillations are the most interesting. They are found both in engineering (e.g., in chemical reactors [1,2,3]) and in such fields as cryptography (e.g., [4]). Research in the field of chaos covers issues such as hyperchaos and multistability [5,6], Lyapunov exponents [7], or systems with lags [8]. One of the aspects of research on chaos is the so-called hidden attractors. Much has been said in the scientific literature about so-called hidden attractors, including hidden stable periodic attractors and hidden chaotic attractors. We deal with a hidden attractor when the mathematical model of a given system does not have a constant dynamic equilibrium point (stationary point). Previous literature reports on this subject concern both dynamic continuous systems (e.g., [9,10,11,12,13,14,15,16,17,18,19,20,21,22,23,24,25]), and discrete multidimensional dynamic systems (for example: [26,27,28,29,30]). Research on this issue is crucial because it can protect the system from dangerous, chaotic oscillations. However, due to their nature, it is difficult to find them at the design stage [31]. Dynamical systems with hidden attractors may also have a positive impact on the development of some fields, for example, in the encryption of images [22,32,33] or in the wireless control of mobile robots [34].

This work presents examples of periodic orbits without an equilibrium point and hidden chaotic attractors in one-dimensional discrete systems. Using Lyapunov’s exponents it was shown that periodic orbits without an equilibrium point are unstable. These orbits are a solution of the multiple compounding of a given discrete transformation, which means that their detection by means of numerical simulation is practically impossible. This problem is significantly simplified when the mathematical model of a given discrete system is an even or at least symmetric function. From the examples presented in this paper, mathematical dependencies were derived that explicitly define the unstable periodic orbits. Hidden chaotic attracting orbits have been determined only numerically. The behavior of partial sums composed of elements of chaotic attractors was also investigated in this work. The fractal nature of these sums was shown, which is presented in the appropriate figure.

The goal of our work is to show that hidden chaotic attractors can occur not only in complex multivariate continuous systems but also in simple single discrete systems that are, for example, described by recursive equations, and that they are quite common in such systems. We also demonstrate repellers in discrete systems without an equilibrium point. The scientific literature mainly deals with hidden attractors in continuous systems. There is, however, a clear deficiency in discrete systems.

In our work, we use tools of nonlinear dynamics, especially chaotic dynamics and fractals. These are primarily the formulas for calculating the Lyapunov exponents, the sensitivity test on the change of initial conditions, and the phase diagram in the form of a discrete fractal map (Figure 5).

This article is structured as follows: The Introduction section provides an overview of the topic and an overview of similar works in the scientific literature; the Mathematical Foundations section describes a method of constructing discrete maps that generate hidden orbits as a result of iteration; in the next part examples of maps constructed with the proposed method are presented; then, in the section devoted to the Euler method, the method of constructing maps on the example of numerical integration is shown. The article ends with conclusions and references.

## 2. Mathematical Foundations

Suppose a function f(x) such that for every real number *x*:

(1) f(x)≠0.

To derive a discrete model without a fixed point, we will use the well-known Newton’s method for determining the roots of the equation. Thus, the following recursive Newton process:

(2) $$x_{k+1}=x_{k}-\frac{f(x_{k})}{f^{\prime }\left(x_{k}\right)}$$

is not a convergent process. This means that the transformation (2) does not have a dynamic equilibrium point (fixed point). In this case, the stable periodic orbits and the chaotic attracting orbits are hidden ones. $F^{\left(m\right)}\left(x\right)$ should be understood as the *m*-th composition of the recursive transform (2) (*m*-th cycle). All *m*-periodic orbits (m>1) can be determined from the *m*-th transformation of the relationship (2):

(3) $$x_{k+m}=F^{\left(m\right)}\left(x_{k}\right).$$

Assuming: $x_{k+m}=x_{k}=x_{sm}$, the *m*-periodic orbit are the roots of the equation (m>1):

(4) $$x_{sm}=F^{\left(m\right)}\left(x_{sm}\right).$$

In case when the function f(x) is even, i.e., f(x)=f(−x), the two-period orbit is defined by the points $x_{s1}=-x_{s2}$. They can be determined directly from (2) as a solution to the equation:

(5) $$-x_{s1}=F^{\left(1\right)}\left(x_{s1}\right).$$

In the case where f(x) is a symmetrical function with respect to $x^{*}\neq 0$, the plot of the function should be shifted so that the zero point becomes the point of symmetry and then formula (5) should be applied. In all other cases, formula (4) should be used to determine other periodic orbits. The other attracting orbits are chaotic orbits.

## 3. Examples

**Example** **1.** *Let us suppose:*

(6) $$f\left(x\right)=x^{2n}+c^{2}=0.$$

*This equation has no solution, and the function f(x) is an even function. Therefore, applying Formula *(5)*, we get that the two-period orbit of transformation *(2)* are the points:*

(7) $$x_{s1,2}=\pm \sqrt[{2n}]{\frac{c^{2}}{4n-1}}.$$

*This orbit is unstable, as evidenced by the value of Lyapunov’s exponent:*

(8) λ=ln[2(2n−1)]>0.

**Figure 1:**
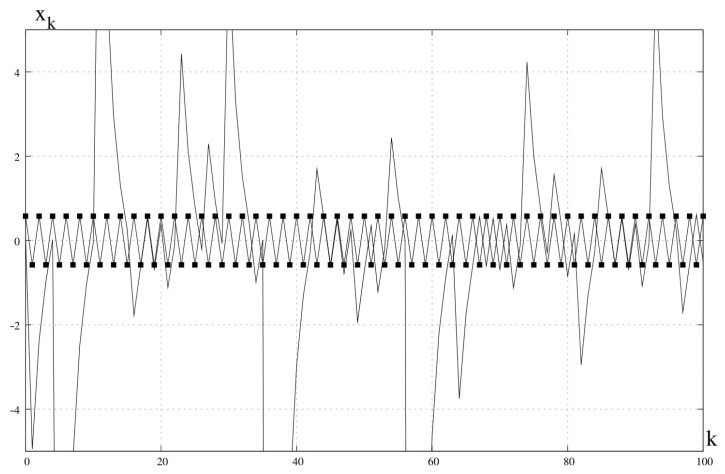
Unstable periodic orbit $\left(x_{s1}=-\frac{1}{\sqrt{3}},\;x_{s2}=\frac{1}{\sqrt{3}}\right)$ and hidden chaotic attractor. n=1, c=1, $x_{0}=0.1$.

*This transformation also has a hidden chaotic attracting orbit (Figure 1).*

**Example** **2.** *Let us suppose:*

(9) $$f\left(x\right)=\exp\left(cx^{2n}\right)=0.$$

*This equation has no solution, and the function f(x) is an even function. Therefore, applying Formula *(5)*, we get that the two-period orbit of transformation *(2)* are the points:*

(10) $$x_{s1,2}=\pm \frac{1}{\sqrt[{2n}]{4nc}}.$$

*This orbit is unstable, as evidenced by the value of Lyapunov’s exponent:*

(11) λ=ln[1+2(2n−1)]>0.

**Figure 2:**
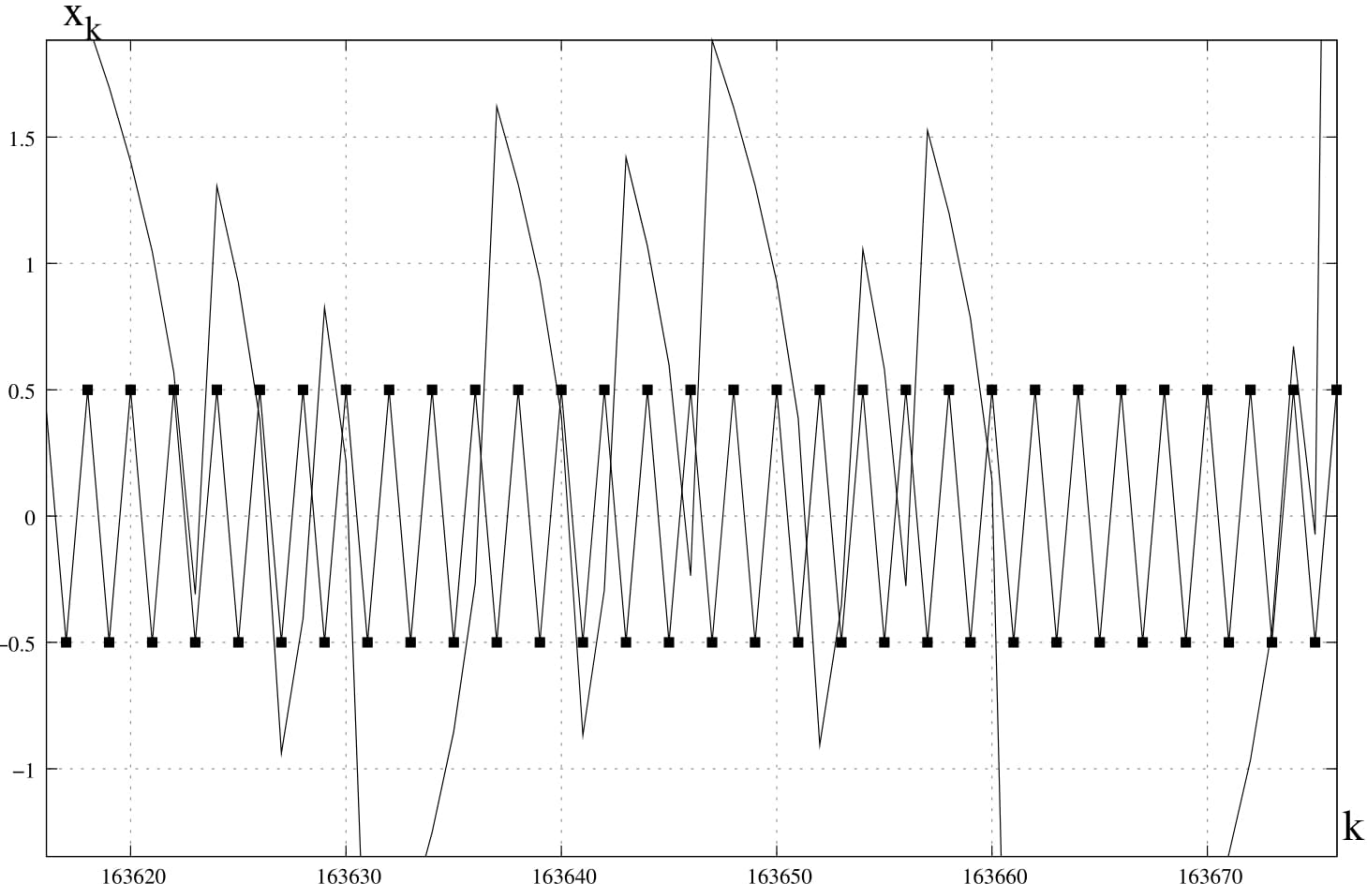
Unstable periodic orbit $\left(x_{s1}=-\frac{1}{2},\;x_{s2}=\frac{1}{2}\right)$ and hidden chaotic attractor. n=1, c=1, $x_{0}=0.1$.

*This transformation also has a hidden chaotic attracting orbit (Figure 2).*

**Example** **3.** *Let us suppose:*

(12) $$f\left(x\right)=x^{2}+x+1=0.$$

This equation has no solution. The function f(x) is not an even function, but it is a symmetrical function with respect to the point $x^{*}=-\frac{1}{2}$. The two-period orbit of Transformation *(2)* is defined in this case by the points:

(13) $$x_{s1}=-1;\;x_{s2}=0.$$

*This orbit is unstable, as evidenced by the value of Lyapunov’s exponent:*

(14) λ=ln2>0.

**Figure 3:**
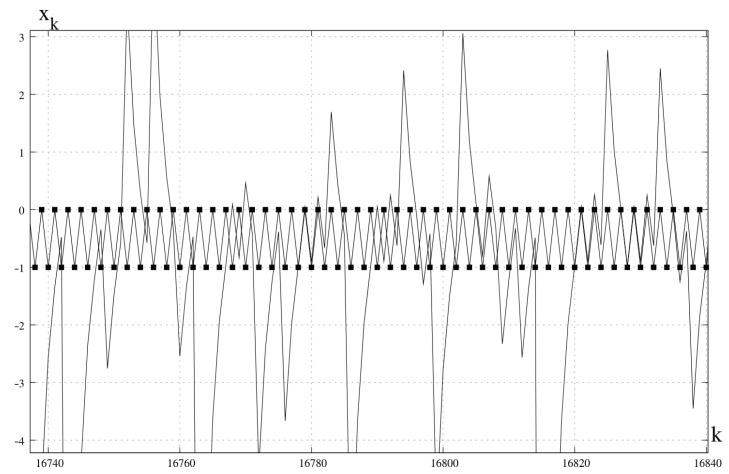
Unstable periodic orbit $\left(x_{s1}=-1,\;x_{s2}=0\right)$ and hidden chaotic attractor. $x_{0}=0.1$.

*This transformation also has a hidden chaotic attracting orbit (Figure 3).*

*The remaining higher-order orbits (m>2), occurring in the above examples, can be determined using Formula* (4)*.*

### Example 4

**Example** **4.** *Let us suppose:*

(15) $$f\left(x\right)=x^{2n}+\exp\left(cx\right)=0.$$

**Figure 4:**
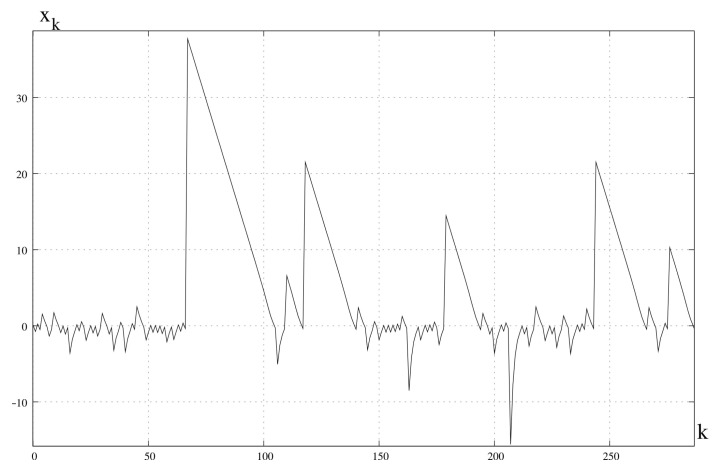
Hidden chaotic attractor. $x_{0}=0.1$.

*This equation has no solution, and the function f(x) is neither even nor symmetric. Transformation (2) based on this function has no periodic orbit, but a chaotic attracting orbit (Figure 4).*

*An interesting issue is to check the behavior of average partial sums, defined as:*

(16) $$S_{k}=\frac{1}{k}\sum_{i=1}^{k}x_{i};\;k=1,2,\cdots$$

**Figure 5:**
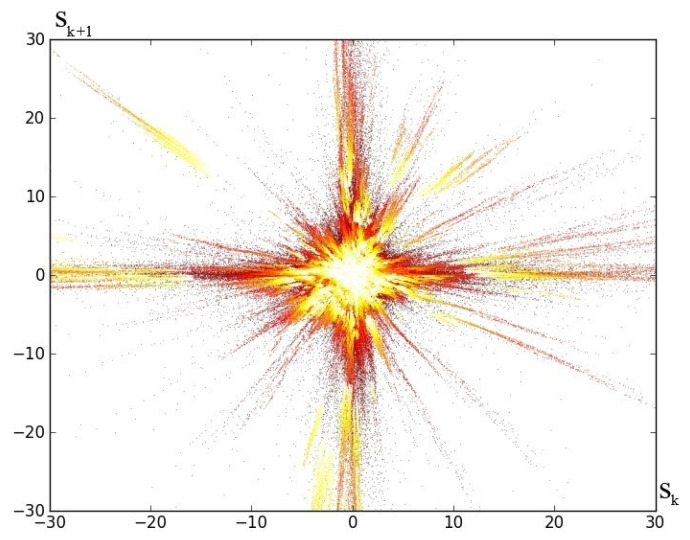
Fractal of partial sums for Example 1.

*Taking into account Example 1, the points with the coordinates $\left(S_{k},S_{k+1}\right)$ are marked in Figure 5.*

*The color of the point depends on the value of k. This graph is a fractal. The drawings made for the remaining examples look similar.*

## 4. Hidden Chaotic Attractors with Euler Integration Method

Suppose we have the following differential equation:

(17) $$\frac{dx}{dt}=g\left(x\right),$$

where g(x)=0 has no solution. Let us say the function g(x) can be represented as:

(18) $$g\left(x\right)=-\frac{f(x)}{f^{\prime }\left(x\right)},$$

which, in conjunction with (17), gives:

(19) $$\frac{dx}{dt}=-\frac{f(x)}{f^{\prime }\left(x\right)},$$

where f(x)=0 has no solution.

Simple transformations of dependencies (19) lead to the general final form:

(20) $$f\left(x\right)=f\left(x_{0}\right)e^{-t},$$

where $x_{0}=x\left(t=0\right)$. It is clearly visible that for t=0, Equation (20) has a solution that is $x=x_{0}$. Therefore, there must be a limit $t_{l}$, above which the equation no longer has a solution. Suppose, for example, that:

(21) $$f\left(x\right)=x^{2}+1,$$

where then:

(22) $$g\left(x\right)=-\frac{x^{2}+1}{2x}.$$

It is thus clear that in this case:

(23) $$t_{l}=\ln\left(x_{0}+1\right).$$

Integrating Equation (17) using the Euler method we obtain the recursion:

(24) $$x_{k+1}=x_{k}-\frac{f(x_{k})}{f^{\prime }\left(x_{k}\right)}\Delta t.$$

**Figure 6:**
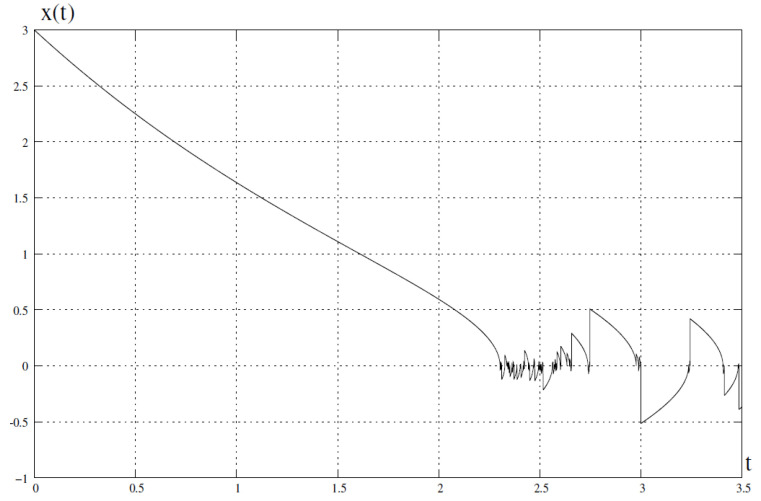
Numerical solution obtained from (24). After reaching $t_{l}=2.3026$ the solution becomes chaotic. $x_{0}=3$.

This means that for $t<t_{l}$ the numerical process tends to a specific value of the variable *x* (for $t=t_{l}\;x=0$), while for $t>t_{l}$ the numerical process becomes chaotic and has the feature of hidden chaos, regardless of the value of the integration variable Δt. This phenomenon is shown in Figure 6 for $x_{0}=3$. The limit value, in this case, is $t_{l}=2.3026$.

## 5. Summary

The examples presented in the paper show the existence of unstable periodic orbits without an equilibrium point and hidden chaotic attracting orbits in discrete models. In the scientific literature, these attracting orbits are called “hidden” because the mathematical models from which they were generated do not have dynamic equilibrium points. Therefore, detecting these types of orbits is extremely difficult. For these examples, mathematical formulas were derived to define unstable periodic orbits without an equilibrium point. Newton’s method and Euler’s method utilized in the work are used to obtain discrete mappings that generate hidden chaotic solutions for appropriately selected functions. The examples presented in the paper show the existence of unstable periodic orbits without an equilibrium point and hidden chaotic orbits in discrete models.
